# Computed Tomography-Based Radiomics Model for Predicting the WHO/ISUP Grade of Clear Cell Renal Cell Carcinoma Preoperatively: A Multicenter Study

**DOI:** 10.3389/fonc.2021.543854

**Published:** 2021-02-25

**Authors:** Ruihui Wang, Zhengyu Hu, Xiaoyong Shen, Qidong Wang, Liang Zhang, Minhong Wang, Zhan Feng, Feng Chen

**Affiliations:** ^1^ Department of Radiology, First Affiliated Hospital, Zhejiang University School of Medicine, Hangzhou, China; ^2^ Department of Radiology, Second People’s Hospital of Yuhang District, Hangzhou, China; ^3^ Department of Radiology, Zhejiang Cancer Hospital, Hangzhou, China; ^4^ Department of Radiology, Yijishan Hospital of Wannan Medical College, Wuhu, China

**Keywords:** computed tomography, multicenter study, WHO pathological grade, radiomic features, radiological model, clear cell renal cell carcinoma (ccRCC)

## Abstract

**Purpose:**

To examine the ability of computed tomography radiomic features in multivariate analysis and construct radiomic model for identification of the the WHO/ISUP pathological grade of clear cell renal cell carcinoma (ccRCC).

**Methods:**

This was a retrospective study using data of four hospitals from January 2018 to August 2019. There were 197 patients with a definitive diagnosis of ccRCC by post-surgery pathology or biopsy. These subjects were divided into the training set (n = 122) and the independent external validation set (n = 75). Two phases of Enhanced CT images (corticomedullary phase, nephrographic phase) of ccRCC were used for whole tumor Volume of interest (VOI) plots. The IBEX radiomic software package in Matlab was used to extract the radiomic features of whole tumor VOI images. Next, the Mann–Whitney U test and minimum redundancy-maximum relevance algorithm(mRMR) was used for feature dimensionality reduction. Next, logistic regression combined with Akaike information criterion was used to select the best prediction model. The performance of the prediction model was assessed in the independent external validation cohorts. Receiver Operating Characteristic curve (ROC) was used to evaluate the discrimination of ccRCC in the training and independent external validation sets.

**Results:**

The logistic regression prediction model constructed with seven radiomic features showed the best performance in identification for WHO/ISUP pathological grades. The Area Under Curve (AUC) of the training set was 0.89, the sensitivity comes to 0.85 and specificity was 0.84. In the independent external validation set, the AUC of the prediction model was 0.81, the sensitivity comes to 0.58, and specificity was 0.95.

**Conclusion:**

A radiological model constructed from CT radiomic features can effectively predict the WHO/ISUP pathological grade of CCRCC tumors and has a certain clinical generalization ability, which provides an effective value for patient prognosis and treatment.

## Introduction

Renal cell carcinoma (RCC) is one of the most common primary malignancies, and clear cell renal cell carcinoma(ccRCC)is the most common subtypes accounting for 60–85% of renal malignancies (1, 2). ccRCC exhibits have high invasive potential. The pathologic nuclear grade of ccRCC is strongly correlated with the 5-year survival rate (3). A higher pathologic nuclear grade implies a worse prognosis. Nuclear grades are an independent prognostic factor for renal tumors (4, 5).

The Fuhrman grading system was the widely used pathology grading system previously, which individual the Fuhrman grade by the cell nucleus size of tumor cells, cell nuclear morphology, and nucleolar prominence. These three parameters are used to classify RCC into four grades (6, 7). However, there has always been a controversy over the Fuhrman grading system. First, this grading system uses three parallel parameters but these parameters may contradict each other in clinical practice. Second, there exists subjective bias on nuclear morphology and nuclear diameter resulting in low repeatability for nuclear grading between pathologists (8, 9).

In order to solve the problems associated with the Furhman grading system, the World Health Organization and International Society of Urological Pathology proposed the WHO/ISUP grading system. This grading system only evaluates nucleolar prominence and classifies tumors into grades I-IV. The determination criteria are simplified and clear, which increases the accuracy of grading kidney cancer (10, 11). Dagher et al. compared the new and old grading system and found that the WHO/ISUP grade is a better independent prognostic factor (12).

Previous ccRCC studies found a correlation between image characterization and Furhman grading (13), but the current clinicopathological nuclear grading criteria have changed, and thus there is a need to reevaluate Radiological studies related to the new grading system. We collected ccRCC image data from many hospitals aimed to create a prediction model based on CT radiomic features with predicting the WHO/ISUP pathological grade of ccRCC. The generalization of the external data build the independent external validation and evaluation model offered preoperative prediction of WHO/ISUP grade and improves patient prognosis.

## Materials and Methods

### Patients

This retrospective study was approved by the Hospital Review Board. The requirement for informed consent was waived. This study included the CT examination of 197 patients with ccRCC confirmed by two pathologists biopsy or surgical resection above four hospitals from January 2018 to August 2019. Of these, 122 patients’ data in the First Hospital of Zhejiang Province were used as the training set, and 75 cases from other three hospitals (Ningbo First Hospital/Zhejiang Cancer Hospital/Yijishan Hospital of Wannan Medical College) were used for external independent external validation (**Table 1**).

**Table 1:** Patient characteristics and image features in the training and validation cohorts.

Characteristic and feature	Training (N = 122)	Validation (N = 75)	*p*-value
Tumor Size (cm)	5.2 ± 1.5	5.3 ± 1.7	0.820
Age			
Range(year)	16–78	18–75	0.364
Sex (number)			0.420
Male	64	48	
Female	58	27	
WHO/ISUP Grade			0.943
Low Grade (I, II)	80	50	
High Grade (III, IV)	42	25	
Location (number)			0.203
Left kindey	74	51	
Right kindey	48	24	
Calcification (number)			1.000
Without	60	37	
With	62	38	
Number of Tumors			0.451
Single	100	65	
Multiple	22	10	

The inclusion criteria were: (1) All patients received enhanced kidneys CT examination before surgical resection including plain scans, corticomedullary phase, and nephrographic phase; (2) There are at least 7 layers in the CT lesion axial image; (3) All tumors underwent surgical resection or percutaneous biopsy and were pathologically confirmed ccRCC; (4) No patients received any treatment before the CT examination. Patients whose image data influenced significantly by artifacts presenced in CT examination were exclusion criteria. In previous studies, WHO/ISUP grades I–II were classified as low-grade and grades III–IV were high-grade.

### CT Technique

CT examtions were obtained from four hospital’s different CT scanners. Patients were given the peripheral intravenous injection of iohexol (300mg/ml non-ionic contrast agent) *via* a high-pressure injector at a flow rate of 2.5–3.0 ml/s and a total dose of 80–100mL (1.0 ml/kg). The scanning range is from the adrenal region to the kidney’s inferior pole. after The corticomedullary phase (CMP) of relative enhanced scan was started 25–28 s after the contrast agent injected from, The enhanced scan for the nephrographic phase (NP) of the kidneys was started 65–70s after intravenous infusion. The CMP and the NP began 25–28 s and 65–70 s after contrast injection, respectively. The scanning and reconstruction parameters of the four CT scanners are shown in **Table 2**.

**Table 2:** The protocols of the CT scan for the patients with a renal mass.

CT scanner	CT256	CT64	CT64	CT320
Scanner model	Brillance-Ict	Revolution EVO	Definition Flash	Aquilion ONE
Manufacturer	Philips	General Electric	SIEMENS	Toshiba
Tube voltage (Kv)	120	120	120	120
Tube current(mAs)	300-350	Automas,300-350	CAREDose4D 350	AIDR 3D 350
Collimation (mm)	128*0.625	64*0.625	64*0.6	160*0.625
Kernel	Stardand(B)	Standard	B30f	Fc10
Slice thickness (mm)	5	5	5	5
Field of view (mm^2^)	350 × 350	350 × 350	350 × 350	350 × 350
Matrix	512 × 512	512 × 512	512 × 512	512 × 512

### Demographic and Clinical Characteristic Analysis

The Chi-square test was used to compare the qualitative variables while the t-test was used for comparison of continuous variables. R software version 3.3.2 (http://www.R-project.org) was used for statistical analysis of the data.

### Process of Radiomics Analysis

The IBEX software package in Matlab was used for tumor separation and extraction of radiomic features (14). We manually outlined the tumor boundaries layer-by-layer in CT images of the CMP and the NP. The first and last layers were discarded, and the remaining layers were combined to obtain the volume of interest (VOI). The lesion boundaries cannot be accurately identified in the tumor boundary and were not used in this study. At the early stage of the study, we randomly selected images from 20 patients and two radiologists with 10 or more years of work experience; independently outlined the VOI. The intra-class correlation coefficient (ICC) was used to evaluate consistency. The VOI extraction of the remaining images was carried out by one radiologist. The features with low repeatability were discarded and features with ICC>0.85 were retained.

The radiomic feature include six major types: intensity histogram, intensity direct, gray level co-occurrence matrix, neighbor intensity difference, gray level run length matrix, morphology and size. The 760 radiomic features were extracted from every VOI. Different computer tomography and scanning parameter will affect the texture parameters. Orlhac et al. proved that the COMBAT compensation algorithm was used to calibrate radiomic data from multiple centers which is entirely data-driven and does not require resampling of CT images in advance (15).

To reduce the number of unrelated and redundant radiomic features, the Mann-Whitney U test was first used on the training set to evaluate the statistical ability of high/low grade for every feature region; features with *p*<0.05 were retained. Next, the minimum redundancy–maximum relevance score (mRMR) was used to sort potential features and obtain the feature subset. Finally, the Akaike information criterion (AIC) was used as a stop criterion and stepwise logistic regression was used to select final features and construct the best radiomic prediction model (16).

### Performance Evaluation

Discrimination, clinical translational value, and calibration were used for detailed evaluation of the prediction model for the training set. Receiver operating characteristic curves (ROC) were used to evaluate the discrimination of the prediction model for low/high ccRCC grade. The decision curve was used to observe whether the model has clinical effectiveness. Next, the model was further valuated by external validation data.

## Results

The baseline characteristics of the patients are shown in **Table 1**. There were no significant statistical differences between the demographic or clinical characteristics between the training set and the independent external validation set (*p* > 0.05). Of the 1520 radiomic features in the CMP and NP phases, 1338 had good repeatability (intraclass correlation coefficient of ≥0.85), and the dimensionality reduction section was based on these features. First, with the minimum redundancy–maximum relevance score (mRMR) algorithm applied, 20 features was used to select the best subset. Second, AIC-based stepwise logistic regression was exploited in further filtering of features. Finally, six features were retained: Three were CMP features, and three were NP features. The feature selection results are summarized in **Table 3**. **Table 3** lists the contribution of every prediction variable in the 2 models and the performance of the model in the training/validation set.

**Table 3:** Risk factors for the differentiation of high from low grade ccRCC.

Variable	Feature Class	Coeffcient	OR (95% CI)	*p* value
Intercept		1.35		0.44
Sum Average (CMP)	Gray Level Cooccurence Matrix (n = 594)	-0.018	0.982(0.953, 1.016)	0.08
Contrast (CMP)	Neighbor Intensity Difference(n = 10)	-0.015	0.984(0.972, 0.998)	0.02
50 Percentile Area(CMP)	Intensity Histogram(n = 48)	-0.15	0.864(0.731, 0.985)	0.04
Local StdMedian (NP)	Intensity Direct(n = 56)	-0.56	0.575(0.513, 0.626)	<0.001
Busyness (NP)	Neighbor Intensity Difference	-1.56	0.213(0.182, 0.245)	0.04
Coarseness (NP)	Neighbor Intensity Difference	1.5	4.486(2.712, 7.347)	0.04

**Figure 1:**
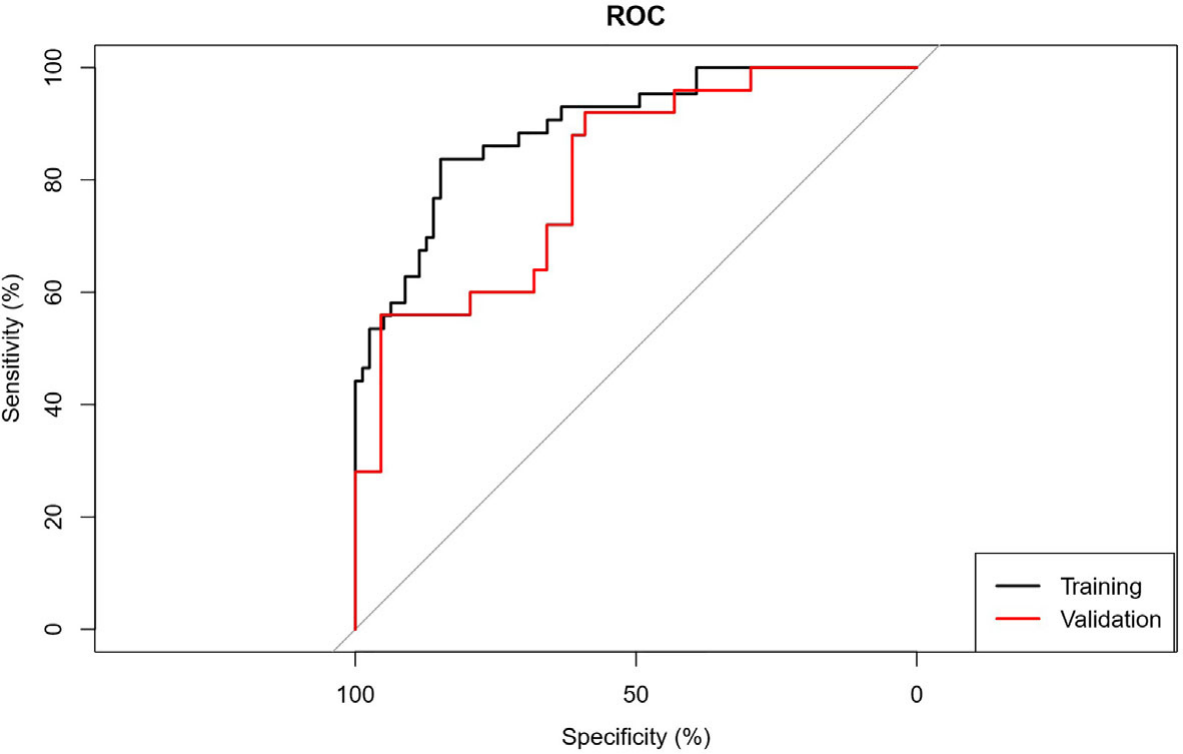
ROC graph. Receiver operating characteristic curves (ROC) were used to evaluate the discrimination of the prediction model for low/high grade CCRCC.

**Figure 2:**
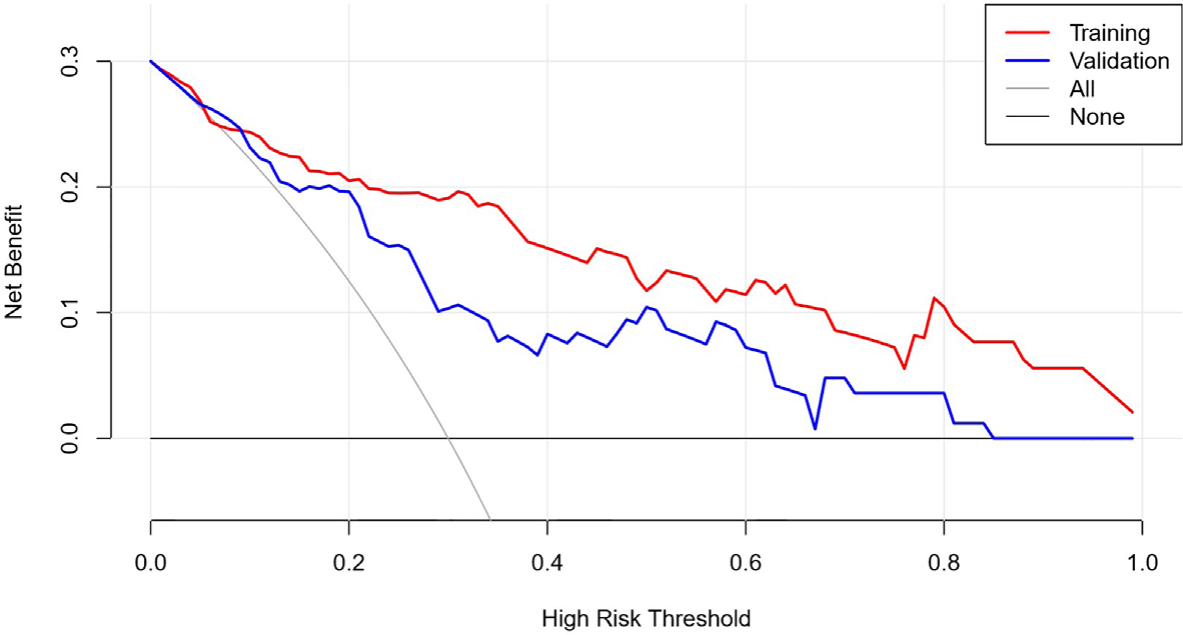
DCA graph. See attached clinical decision curve: training set validation set, the decision curve was used to observe whether the model has clinical effectiveness.

**Figure 3:**
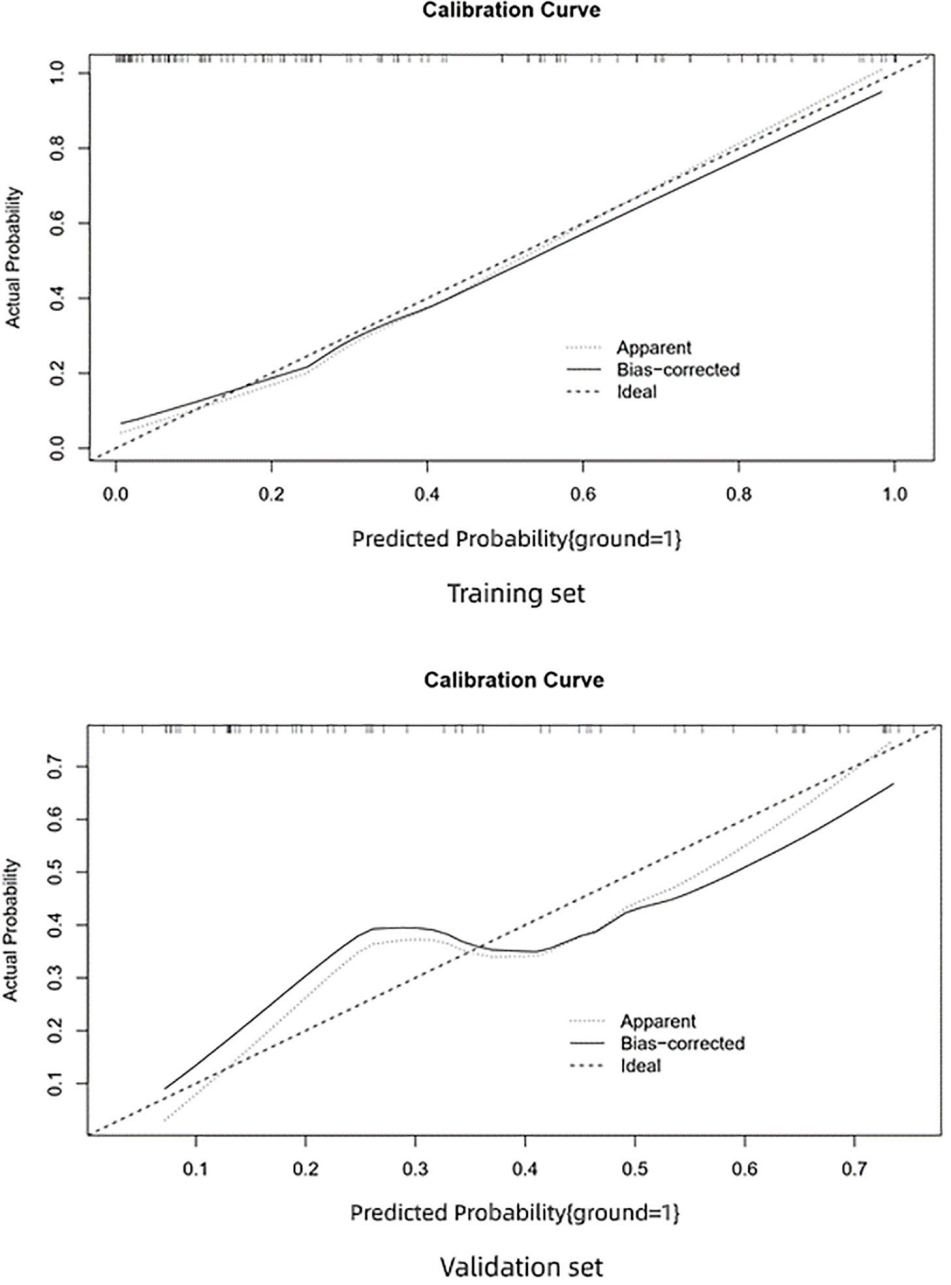
Calibration curve. Calibration data show the relationship between predicted risk and actual risk.

The AUC of the prediction model in the training set was 0.89, sensitivity was 0.85, and specificity was 0.84. In the independent external validation set, the AUC of the prediction model was 0.81, sensitivity was 0.58, and specificity was 0.95, discrimination was a bit decreased versus the training set (**Figure 1**). As shown in **Figure 2**, the decision curves of the predictive model in the training and independent external validation sets. The **Figure 3** shows that the predictive model has good clinical net benefit threshold probabilities of 10–100% in the training set. In the independent external validation set, the clinical net benefit range has threshold probabilities of 10–85%. In addition, the net benefit of the training set model was higher than the independent external validation set.

The calibration curve of the training set shows very good consistency between the prediction probability and observed frequency. The goodness of fit between the prediction probability and observed frequency in the calibration curve of the validation set is not as good as the training set. The prediction model shows good prediction performance in the training and validation sets. However, in comparison, the prediction performance of the training set was better and performance decreased in the independent external validation set.

## Discussion

We constructed new CT radiomic prediction models for new ccRCC pathologic nuclear grades. The model not only demonstrates outstanding ability to discriminate low/high WHO/ISUP grades in the training set but also offered good performance in the external independent test data at the same time. Many past studies demonstrate that imaging characteristics have potential value in distinguishing Fuhrman grades. Zhu et al. found that low enhancement at the CMP is an independent predictor for high-grade tumors. Huhdanpaa et al. (17) found that the interquartile range of histogram parameters at the NP can distinguish low/high Fuhrman grades. Radiomic studies employ and screen image feature parameters, and use machine learning algorithms to construct nuclear grade classification models. The results of these studies are better than early researchs. Shu et al. (18) employed radiomics for Fuhrman grade prediction to set a CMP radiomic model, a NP phase radiomic model, and a combination of the two phases, the result of AUC was 0.77, 0.81, and 0.82, respectively. Ding et al. (19) find when only texture parameters were used in Fuhrman grade prediction, the original of AUC comes to 0.84, and that increased to 0.87 after some non-texture parameters were added. Good results were shown in these prior studies. However, due to the the classification confusion stardand, we cannot avoid the reality that the Fuhrman grading system has been abandoned in clinical practice.

Studies based on the new WHO/ISUP grading system will undoubtedly have important practical and clinical significance. Currently there are relatively few radiological studies based on the WHO/ISUP grading system: Sun et al. (20) similarly used a combined the CMP and the NP phase model to predict the WHO/ISUP pathological grade. The highest AUC was 0.88 while sensitivity and specificity were 0.83 and 0.89, respectively. Shu et al. also simultaneously compared the performance of multiple machine learning algorithms in predicting the WHO/ISUP grade; the AUC basically remained above 0.90. Our results are similar to other studies while the performance of radiomic models for WHO/ISUP grading is slightly better than the previous Fuhrman grading results. This may be related to the more accurate WHO/ISUP grading Indeed, in our case review of ccRCC patients, we often encounter inaccurate Fuhrman grades such as pathological reports of Fuhrman II or Fuhrman III grades. These ambiguous results will inevitably lead to problems in studies on Fuhrman grades.

We note that many past nuclear grade radiomic studies only offered internal independent external validation in which data were simply divided into a 7:3 ratio, into a training set and validation set; all data were obtained from a single instrument in a single center. The good results were only based on a single center’s data for ignoring the acquisition parameters in varying degrees always affect radiomic features. Therefore, these models will inevitably have different degrees of overfitting. Thus, a single-center study has limitations, and an independent external validation data is required for predictive models that accurately evaluate generalization.

The strength of this study is data from three other hospitals were collected to construct the independent validation dataset. The AUC of the predictive model in independent external validation decreased, but the decrease is small; the AUC was still 0.80 with a good model performance. The independent external validation decreased to 0.58, We speculated that there are differences in the data from the three hospitals, and the ratios of low/high grades in the data are not identical. This can decrease the independent external validation performance. However, this fits closer to actual clinical practice data and shows that the predictive model in this study can be generalized.

The early diagnosis rate of ccRCC has been significantly improved, but a kidney cancer patient with tumor diameter <4 cm may have potential metastasis at initial diagnosis. Even if radical nephrectomy or partial nephrectomy was carried out in early stage kidney cancer, 20–30% of patients still develop local or distal metastasis. The pathologic nuclear grade of ccRCC is correlated with metastatic potential and affects patient prognosis. Therefore, the early prediction of the nuclear grade is extremely important which is of great significance for clinical decisions and improving the long-term survival and quality-of-life.

This study has several limitations: (1) Although independent external validation was carried out, the sample size was still relatively small and the sensitivity of the prediction model was relatively low. The reason may be mainly attributed to the fact that our external validation set is actually a combination of different data from three different hospitals acquired with different equipment. Therefore, it is understandable that the radiomics parameters may vary to some extent. Although the COMBAT algorithm was used to correct the data, the ability of this algorithm may still not strong enough to overcome the data variation. (2) The predictive model in this study was limited to only distinguish high/low-grade ccRCC. However, in clinical practice, it is more important to identify the malignancy of RCC. (3) We did not include subjective image features as they are affected by the experience of radiologists. We also did not include the clinical characteristics in our model. The main reason may due to that several studies have indicated the relatively low specificity of clinical features in predicting the grade of CCRCC. (4) Our study did not include plain CT scans because, it is difficult to identify the boundaries of certain ccRCC tumors based on experience. However, some reports claimed that plain CT texture analysis can still be used to predict the nuclear grade of CCRCC. We believe in the future, there will be new semi-automated software identify RCC boundaries.

## Conclusion

In the era of precision medicine, nuclear grade prediction will aid in clinical decision-making and prognosis. Multicenter internal/external validation proved that CT radiomics can accurately predict the WHO/ISUP grade which means the CT radiomic prediction model can be used as an auxiliary tool for prediction of the WHO/ISUP grade in ccRCC and aid in personalized treatment.

## Data Availability Statement

The original contributions presented in the study are included in the article/supplementary material. Further inquiries can be directed to the corresponding authors.

## Ethics Statement

Ethical approval was obtained from the Human Research Ethics Committee (HREC) of First Affiliated Hospital of Zhejiang University School of Medicine. The patient informed consent was waived by the HREC for the retrospective usage of patients’ medical images.

## Author Contributions

FC, ZF, XS, and QW conceived the project. RW and ZH analyzed the data and wrote the paper. LZ and MW collected the data. All authors contributed to the article and approved the submitted version.

## Funding

This study has received funding by the Department of Health of Zhejiang Province China (no. 2019KY551).

## Conflict of Interest

The authors declare that the research was conducted in the absence of any commercial or financial relationships that could be construed as a potential conflict of interest.
